# Blind Spot in the Radar of MEST-C Score: Type and Severity of Tubulointerstitial Nephritis in IgA Nephropathy

**DOI:** 10.1155/2023/1060526

**Published:** 2023-03-13

**Authors:** Iram Asrar, Mudassar Hussain, Aurangzeb Afzal, Usman Hassan, Sheeba Ishtiaq

**Affiliations:** ^1^Department of Pathology, Shaukat Khanum Memorial Cancer Hospital and Research Centre, Lahore, Pakistan; ^2^Department of Nephrology, Services Hospital, Lahore, Pakistan; ^3^Department of Pathology, Gulab Devi Chest Hospital, Lahore, Pakistan

## Abstract

**Background:**

The updated version of predictive classification for immunoglobulin A nephropathy (IgAN) prognosis “The Oxford Classification” identifies five histopathological features including mesangial hypercellularity (M), endocapillary proliferation (E), segmental glomerulosclerosis (S), tubular atrophy/interstitial fibrosis (T) and crescents (C), the MEST-C. However, few studies suggest that tubulointerstitial inflammation, which is not included in the MEST-C, is also linked to disease progression and is, consequently, a neglected determinant of prognosis among others. Therefore, there is a need to evaluate this histopathological parameter in patients with IgA nephropathy.

**Materials and Methods:**

This cross-sectional descriptive study was conducted at Shaukat Khanum Memorial Cancer Hospital and Research Center, Lahore, Pakistan. Data of histopathological and immunofluorescence proven renal biopsies (300) of IgA nephropathy patients from January 2016 through May 2022 were extracted using a convenient sampling technique. Biopsies were histologically reviewed for type and severity of tubulointerstitial inflammation, in addition to the MEST-C score. Renal biopsies of patients who had a history of transplant, autolyzed tissue, no glomeruli on histological examination, and/or a tubular atrophy/interstitial fibrosis score of 2 (T2) in MEST-C scoring were excluded. Data were analyzed using SPSS 20. An association between the variables was analyzed using the chi-square and Fischer exact tests. A *p* value less than 0.05 was considered statistically significant.

**Results:**

A total of 247/300 biopsies were eligible for inclusion. The mean age at the time of biopsy was 31.90 ± 12.48 with 63.6% in the age group between 21 and 40 years, and 69.6% were male. Tubulointerstitial inflammation was observed in 90.2% cases with 49.4% showing moderate while 4.5% showing severe degree of inflammation. A strong association of both the type and severity of tubulointerstitial inflammation was found with M, E, T, and C scores (*p* value < 0.05).

**Conclusion:**

The high-frequency and strong statistical association of tubulointerstitial inflammation with the M, E, T, and C scores in our study elucidate its prognostic role in the progression and management of IgA nephropathy.

## 1. Introduction

Immunoglobulin A nephropathy (IgAN), first described by Jean Berger in 1968, is the most commonly recognized primary glomerulonephritis worldwide. It has heterogeneous pathological and clinical presentations, leading variably to end-stage renal disease. It has been known to occur in all age groups and ethnicities [1, 2]. In order to determine and implement an earlier precise treatment intervention, it is imperative to accurately recognize the clinical and pathological findings and predict the prognosis. For this purpose, various classification systems have been suggested over the years. One such predictive histopathological classification for IgAN prognosis was published in 2009: The Oxford Classification. This classification has been validated in different ethnicities to predict prognosis independent of the clinical features, and the updated version identifies five histopathological features including mesangial hypercellularity (M), endocapillary proliferation (E), segmental glomerulosclerosis (S), tubular atrophy/interstitial fibrosis (T), crescents (C), the MEST-C. The MEST-C score has been studied and authenticated in both the pediatric and adult study groups and has assisted nephrologists to better evaluate the course of end-stage renal disease. It was established that the Oxford classification may also help in risk stratification and recruitment of patients into clinical trials for immunosuppression and other specific treatments based on the identification of more specific pathological features [3–6].

In this context, few studies suggest that tubulointerstitial inflammation, which is not included in the MEST-C score, is a specific pathological feature that is linked to disease progression and is, consequently, a neglected determinant of prognosis among other well-known histological features. It is suggested that parameters related to tubulointerstitial inflammation predict a decline in renal function in IgA nephropathy patients [7, 8]. In this regard, the immunohistochemical expression of tubulointerstitial LCA, CD3+T-lymphocytes, CD68-positive macrophages, and IL-1*β* has been studied and associated with the outcome. By establishing the MEST-C score in addition to the type and severity of tubulointerstitial inflammation in renal biopsies of patients with IgAN, we can alarm and guide the clinician to accurately target the medical treatment to obtain significant benefit for such patients [8]. Therefore, there is a need to evaluate this histopathological parameter in renal biopsies of different populations with IgA nephropathy. In this article, we report the frequency and types of tubulointerstitial inflammation in addition to the MEST-C score that we encounter in patients with IgA nephropathy and discuss their clinical relevance in conjunction with the established MEST- C score.

## 2. Materials and Methods

Patients with a renal biopsy diagnosis of IgAN between January 2016 and May 2022 were identified from the archives of Shaukat Khanum Cancer Hospital and Research Center. These patients were diagnosed on the basis of histopathological and immunofluorescence features. This hospital has been serving a wide population of all provinces of Pakistan as well as a small proportion of Afghanis. The available clinicopathological data was recorded from the biopsy reports including age and gender and reviewed for cumulative MEST-C scores, in addition to the type and severity of tubulointerstitial inflammation. The relevant clinical laboratory and follow-up data for most of the patients were unavailable or incomplete and therefore not included in the study. Patients with a history of renal transplant, autolyzed tissue biopsies, and biopsies without glomeruli were excluded. In addition, patients with a T2 score were also excluded so that tubulointerstitial inflammation could be evaluated in an area of viable cortex. The biopsies were reported by two pathologists. The MEST-C score was evaluated according to the international guidelines (Table 1) [2, 4, 9]. Tubulointerstitial inflammation was estimated in the viable cortex by the microscopic presence of acute and chronic inflammatory cells (determined at 10x) in the interstitium and was categorized as absent, acute, acute and chronic, and chronic. The severity of inflammation was considered as mild, moderate, and severe by using the Banff criteria (mild 0–25%, moderate 26–50%, and severe >50%). A viable cortical area was obtained by subtracting the atrophic cortex from the total surface area of the biopsy with tubulointerstitial inflammation.

After collection, the data were arranged and entered for analysis into computerized software called Statistical Package for Social Sciences (SPSS 20.0). Frequencies and percentages were presented as tables for both qualitative and quantitative variables. Clinicopathological parameters were associated with the type and degree of tubulointerstitial inflammation using the chi-square test and Fischer's exact test. A *p* value <0.05 was considered statistically significant.

## 3. Results

**Figure 1:**
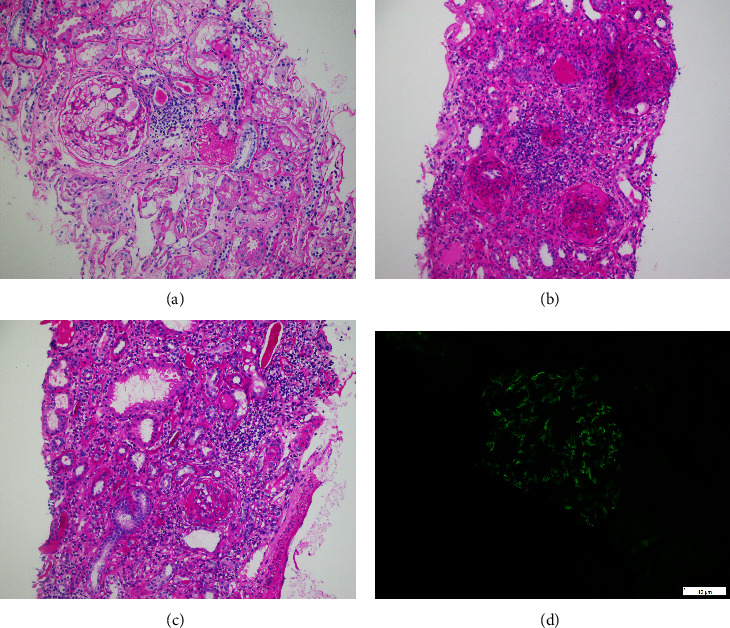
Severity of tubulointerstitial inflammation (periodic acid Schiff stain) and IgA immunofluorescence, 10x. Mild inflammation (a), moderate inflammation (b), severe inflammation (c), and IgA immunofluorescence, mesangial staining (d).

A total of 300 cases of biopsy-proven IgA nephropathy were identified. Of these, 53 cases having tubular atrophy/interstitial fibrosis (T) involving more than 50% cortical area (T2) were excluded. Of the remaining 247 cases, the mean age at the time of biopsy was 31.90 ± 12.48 with 63.6% in the age group between 21 and 40 years and 69.6% were male. Global sclerosis of the glomeruli was observed in 157 cases. Fibrous crescents were observed in 14, cellular crescents in 41, and fibrocellular crescents in 43 cases. The frequencies of MEST-C score along with the type and degree of tubulointerstitial inflammation are given in Table 2. The relationship between the type and degree of tubulointerstitial inflammation and clinicopathological parameters is shown in Tables 3–5, respectively. Tubulointerstitial inflammation was found in 90.2% of cases. It was found that a significant percentage of patients (49.4%) showed a moderate degree of tubulointerstitial inflammation at the time of diagnosis. A strong association was found between the type and degree of tubulointerstitial inflammation with the M, E, T, and C scores (*p* value less than 0.05). The severity of inflammation and IgA immunofluorescence is depicted in Figures 1(a)–1(d).

## 4. Discussion

IgA nephropathy is the most common form of glomerulonephritis worldwide, yet the management of this disease remains challenging due to extensive variation in clinical practice and conflicting data from published literature regarding the use of immunosuppressive therapy. Recognizing the pathological correlates of IgA nephropathy is critical for directing and planning prospective therapies so that maximum patients can obtain benefit. IgA nephropathy has a heterogeneous pathogenesis based on various clinical and pathological factors. It is credible to divulge that virtually no single pathway has a dominant role during disease progression, and diversity is also partly attributable to the genetics of various ethnicities. A few review articles summarize clinicopathological prognostic factors for IgAN. Histopathological parameters like widespread global and/or segmental glomerulosclerosis and marked tubulointerstitial lesions are strong predictors of disease progression [2, 10]. The Oxford Classification has proved to be a remarkable achievement for laying down the foundation to predict the prognosis of this diverse disease and the efforts of researchers are admirable that they continue to evolve it. Tubulointerstitial inflammation is not included in the MEST-C score in Oxford classification. However, it has been studied to be associated with disease progression. A review of the published literature reveals that this was the first study in Pakistan which was conducted on tubulointerstitial inflammation in patients with IgA nephropathy until present. In this study, we specifically observed tubulointerstitial inflammation in addition to the MEST-C score to observe the status of these parameters in IgAN patients in our population.

In addition to glomerular lesions, tubulointerstitial changes such as inflammation were also seen in IgAN renal biopsies. Inflammatory cell infiltrates are considered to reflect an active disease process. However, the type and severity of tubulointerstitial inflammation have not been contemplated much as compared to the MEST-C score [8]. It was previously not included in the classification on the basis of the fact that inflammation in the total cortex was closely correlated with the degree of interstitial fibrosis. In our study, 223 of 247 cases (90.2%) revealed tubulointerstitial inflammation. Of these, 122 (49.4%) cases revealed moderate and 11 (4.5%) cases showed severe degree of inflammation. The inflammatory cell infiltrate comprised of both acute and chronic types. Inflammation was exclusively studied in an area of nonscarred/viable cortex, similar to the reported pattern of transplant biopsies. In this regard, patients with a T2 score were specifically excluded. Frees et al. reported cellular infiltrates in 89% of cases of IgAN, which were described as strong in 8 of these cases and predominantly comprised of lymphocytes alone or mixed with plasma cells. They found an association between these infiltrates with an increased risk and a shorter progression period to renal failure in such patients [11]. Myllymäki et al. studied the parameters reflecting tubulointerstitial inflammation. They reported interstitial inflammation in 25% of the cases and graded inflammation as normal, mild, or marked. They studied the quantitative assessment of inflammation using immunohistochemical expression of LCA, CD3, CD68, and IL-*β* in 204 patients and concluded that evaluation of the level of inflammation and the grade of inflammation can predict the renal outcome [8]. Both of the above-mentioned studies did not describe the area of cortex where inflammation was reported. However, Rankin et al. demonstrated the assessment of active tubulointerstitial inflammation (ATIN) in nonscarred cortex by subtracting the cortex with tubular atrophy from the total area of the cortex harboring interstitial inflammation. This overcame the correlation with interstitial fibrosis. They reported ATIN in 49% cases. They concluded that the presence of more than 10% of active tubulointerstitial inflammation in nonscarred cortex was significantly associated with disease prognosis and correlated independently with prognosis as equal, or even better than the MEST-C score [7].

MEST-C parameters in the Oxford classification have been studied and validated previously for their prognostic role in IgA nephropathy. Mesangial hypercellularity, segmental glomerulosclerosis, tubulointerstitial lesions, particularly T1 and T2, and crescents are associated strongly with disease progression independent of laboratory and clinical parameters, and the predictive role of endocapillary lesions has been linked to immunosuppressive therapy [3, 6, 12]. Haas et al. reported that using corticosteroids can reverse the active lesions and improve the clinical picture [13]. However, the tubulointerstitial lesions (tubular atrophy and interstitial fibrosis) are not improved after immunosuppressive therapy [14]. Results of our histopathological study show that different types of inflammation with variable degrees were observed in 87.8% cases showing M1 score, 47.3% E1, 81.78% S1, 50.2% T1, and 100% of C1 and C2 scores. In our study, tubulointerstitial inflammation showed an association with the M, E, T and C score (*p* value <0.05). A strong association was shown with the E, T, and C scores (p value less than 0.01). These results are comparable to the study conducted by Rankin et al. showing ATIN in 70% M1, 72% E1, 63% S1, 30% T1, 20% C1, and 9% C2 cases (*p* value <0.05) [7]. Inflammation was more pronounced in our renal biopsies. This can be attributed to the lower socioeconomic status, higher illiteracy rate of our population, drug abuse and lack of awareness that these patients present late during disease. This may also be related to the findings of a study conducted by Barbour et al. (2013) that Asian population has a higher risk of progression to end-stage renal disease [15]. However, for unexplainable reasons, no association was found with segmental glomerulosclerosis (*p* value >0.05), similar to the results shown by Rankin et al. This is contrary to the results of other studies which validate the role of segmental glomerulosclerosis in renal outcome [2, 3]. The association of tubulointerstitial inflammation with M, E, T, and C scores suggests that tubulointerstitial inflammation is also linked to disease progression and may help in prediction of the disease progression and survival and hence guide therapy.

The major limitation of our study was a deficiency of clinical laboratory data and follow-up of IgA patients. Therefore, only histopathological parameters were studied. Unfortunately, the capacity for risk stratification into prognostic groups based on known predictive and prognostic factors in our health care system is too bare at present and therefore remains a therapeutic challenge. There is a lack of education and counselling among the patients who are also lost to follow-up. Patients destined to do well without immunosuppressive therapy might receive toxic therapies, and many other patients eventually develop end-stage kidney disease regardless of meticulous therapy. Research studies committed thus far have been impeded by variable evaluation techniques, small sample sizes, and a lack of clinical data or follow-up. For this purpose, it is imperative to create awareness among the patients as well as the clinicians in developing countries. In subsequent years, it is suggested for the clinicians to work in collaboration with the pathologists and formulate an improved standardized approach to evaluate potentially useful factors. A comprehensive approach to disease evolution will lead to robust management of this disease.

Heterogeneity in IgA nephropathy has both spatial and temporal ingenious facets that may be more diverse than is understood at present. Considering the vigorous studies conducted to validate the Oxford classification system, it is disappointing that an important histological parameter like tubulointertstitial inflammation remains a blind spot. It is reasonable to evaluate it further in risk stratification, clinical trials, and follow-up studies to guide management. Tubulointerstitial inflammation in addition to the MEST-C score may behold future refulgence in the management of IgA patients.

## Figures and Tables

**Table 1:** MEST-C criteria of IgA nephropathy [2, 4, 9].

Histological parameter	Definition	Score
Mesangial hypercellularity (M)	Increased number of mesangial cells per unit of mesangium (traditionally more than 3 cells)	(i) M0: <50% of glomeruli showing mesangial hypercellularity (ii) M1: >50% of glomeruli showing mesangial hypercellularity
Endocapillary hypercellularity (E)	An increased number of cells within glomerular capillary lumina causing narrowing	(i) E0: no endocapillary hypercellularity (ii) E1: any glomeruli showing endocapillary hypercellularity
Segmental glomerulosclerosis (S)	Obliteration of capillary lumina by fibrosis in less than 50% of the glomerular tuft	(i) S0: absent (ii) S1: present in any glomeruli
Tubular atrophy/interstitial fibrosis (T)	Estimated percentage of cortical area showing atrophic tubules or interstitial fibrosis, whichever is greater	(i) T0: 0–25% of cortical area (ii) T1: 26–50% of cortical area (iii) T2: >50% of cortical area
Cellular or fibrocellular crescents (C)	Percentage of glomeruli with cellular or fibrocellular crescents; crescent is defined as extracapillary proliferation which can be Cellular: cells more than 2 cells thickness Fibrous: cellular proliferation with increased matrix (<90%), Fibrocellular: proliferation with more than 90% matrix	(i) C0: absent (ii) C1: 0–25% of glomeruli (iii) C2: ≥25% of glomeruli

**Table 2:** Frequency and percentages of histological parameters.

Histological parameters		Frequency	Percentage
Mesangial hypercellularity	M0	9	3.6
	M1	238	96.4

Endocapillary hypercellularity	E0	126	51.0
	E1	121	49.0

Segmental glomerulosclerosis	S0	27	10.9
	S1	220	89.1

Tubular atrophy and interstitial fibrosis	T0	123	49.8
	T1	124	50.2

Crescents (C) score	C0	168	68.0
	C1	59	23.9
	C2	20	8.1

Type of tubulointerstitial inflammation	Absent	24	9.7
	Acute	4	1.6
	Acute and chronic	27	10.9
	Chronic	192	77.7

Degree of tubulointerstitial inflammation	Absent	24	9.7
	Mild	90	36.4
	Moderate	122	49.4
	Severe	11	4.5

Total		247	100.0

**Table 3:** Relationship between the type and degree of tubulointerstitial inflammation.

		Degree of tubulointerstitial inflammation				Total
		Absent	Mild	Moderate	Severe	
Type of tubulointerstitial inflammation	Absent	24	0	0	0	24
	Acute	0	2	2	0	4
	Acute and chronic	0	6	17	4	27
	Chronic	0	82	103	7	192

Total		24	90	122	11	247

*p* value: ≤0.001.

**Table 4:** Relationship between MEST-C score and type of tubulointerstitial inflammation.

Clinicopathological parameters		Type of tubulointerstitial inflammation				*p* value
		Absent	Acute	Acute and chronic	Chronic	
Age	0–20	3	0	6	33	0.49
	21–40	20	3	18	116	
	41–60	1	1	3	39	
	61–80	0	0	0	4	

Gender	Male	13	2	19	138	0.27
	Female	11	2	8	54	

Mesangial hypercellularity	M0	3	0	3	3	**0.008**
	M1	21	4	24	189	

Endocapillary hypercellularity	E0	20	2	10	94	**≤0.001**
	E1	4	2	17	98	

Segmental glomerulosclerosis	S0	6	0	3	18	0.168
	S1	18	4	24	174	

Tubular atrophy and interstitial fibrosis	T0	24	4	12	83	**≤0.001**
	T1	0	0	15	109	

Crescents (C) score	C0	24	3	15	126	**0.003**
	C1	0	0	9	50	
	C2	0	1	3	16	

Total cases (*n*) = 247. Bold *p* values are statistically significant.

**Table 5:** Relationship between MEST-C score and the degree of tubulointerstitial inflammation.

		Degree of tubulointerstitial inflammation				*p* value
		Absent	Mild	Moderate	Severe	
Age	0–20	3	15	22	2	0.77
	21–40	20	55	75	7	
	41–60	1	18	23	2	
	61–80	0	2	2	0	

Gender	Male	13	58	96	5	0.09
	Female	11	32	26	6	

Mesangial hypercellularity	M0	3	5	1	0	**0.023**
	M1	21	85	121	11	

Endocapillary hypercellularity	E0	20	53	48	5	**≤0.001**
	E1	4	37	74	6	

Segmental glomerulosclerosis	S0	6	11	9	1	0.08
	S1	18	79	113	10	

Tubular atrophy and interstitial fibrosis	T0	24	67	31	1	**≤0.001**
	T1	0	23	91	10	

Crescents (C) score	C0	24	74	65	5	**≤0.001**
	C1	0	14	40	5	
	C2	0	2	17	1	

Total cases (*n*) = 247. Bold values are statistically significant.

## Data Availability

The patient data used to support the findings of this study are restricted by the Institutional Review Board of Shaukat Khanum Memorial Cancer Hospital and Research Center in order to protect patient privacy. The data are available from the corresponding author upon request, for researchers who meet the criteria for access to confidential data.

## References

[1] Moriyama T. (2019). Clinical and histological features and therapeutic strategies for IgA nephropathy. *Clinical and Experimental Nephrology*.

[2] Hassler J. R. (2020). IgA nephropathy: a brief review. *Seminars in Diagnostic Pathology*.

[3] Lv J., Shi S., Xu D. (2013). Evaluation of the Oxford Classification of IgA nephropathy: a systematic review and meta-analysis. *American Journal of Kidney Diseases*.

[4] Cattran D. C., Troyanov S., Cook H. T. (2009). The Oxford classification of IgA nephropathy: rationale, clinicopathological correlations, and classification. *Kidney International*.

[5] Trimarchi H., Barratt J., Cattran D. C. (2017). Oxford classification of IgA nephropathy 2016: an update from the IgA nephropathy classification working group. *Kidney International*.

[6] Barbour S. J., Espino-Hernandez G., Reich H. N. (2016). The MEST score provides earlier risk prediction in lgA nephropathy. *Kidney International*.

[7] Rankin A. J., Kipgen D., Geddes C. C. (2019). Assessment of active tubulointerstitial nephritis in non-scarred renal cortex improves prediction of renal outcomes in patients with IgA nephropathy. *Clinical kidney journal*.

[8] Myllymäki J., Honkanen T., Syrjänen J. (2007). Severity of tubulointerstitial inflammation and prognosis in immunoglobulin A nephropathy. *Kidney International*.

[9] Markowitz G. (2017). Updated Oxford classification of IgA nephropathy: a new MEST-C score. *Nature Reviews Nephrology*.

[10] DAmico G. (2004). Natural history of idiopathic IgA nephropathy and factors predictive of disease outcome. *Seminars in Nephrology*.

[11] Freese P., Nordén G., Nyberg G. (1998). Morphologic high-risk factors in IgA nephropathy. *Nephron*.

[12] Rui Y., Yang Z., Zhai Z., Zhao C., Tang L. (2022). The predictive value of Oxford MEST-C classification to immunosuppressive therapy of IgA nephropathy. *International Urology and Nephrology*.

[13] Haas M., Verhave J. C., Liu Z.-H. (2017). A multicenter study of the predictive value of crescents in IgA nephropathy. *Journal of the American Society of Nephrology*.

[14] Shen X. H., Liang S. S., Chen H. M. (2015). Reversal of active glomerular lesions after immunosuppressive therapy in patients with IgA nephropathy: a repeat-biopsy based observation. *Journal of Nephrology*.

[15] Barbour S. J., Cattran D. C., Kim S. J. (2013). Individuals of Pacific Asian origin with IgA nephropathy have an increased risk of progression to end-stage renal disease. *Kidney International*.

